# Dosimetric Comparison of Three Radiotherapy Techniques in Irradiation of Left-Sided Breast Cancer Patients after Radical Mastectomy

**DOI:** 10.1155/2020/7131590

**Published:** 2020-03-26

**Authors:** Jian Hu, Guang Han, Yu Lei, Ximing Xu, Wei Ge, Changli Ruan, Sheng Chang, Aihua Zhang, Xiangpan Li

**Affiliations:** ^1^Department of Radiation Oncology, Wuhan University, Renmin Hospital, Wuhan, 430060 Hubei Province, China; ^2^Department of Radiation Oncology, Hubei Cancer Hospital, Tongji Medical College, Huazhong University of Science and Technology, Wuhan, 430079 Hubei Province, China; ^3^Department of Radiation Oncology, University of Nebraska Medical Center, Omaha, USA; ^4^Department of Oncology, Wuhan University, Renmin Hospital, Wuhan, 430060 Hubei Province, China

## Abstract

**Results:**

The VMAT plans showed superior to PTV dose conformity index (CI), homogeneity index (HI), protection of the ipsilateral lung, monitor units (MUs), and maximum dose (D_max_) to the contralateral breast compared with TSP and 9FIMRT plans. The TSP provided better protection for D_mean_ of the heart and left ventricle (*p* < 0.05). A dose for left anterior descending artery from the three techniques had no significant difference. Compared with the 9FIMRT plans, the V_5Gy_ (%) and V_10Gy_ (%) for the ipsilateral lung were significantly reduced with TSP and VMAT (*p* < 0.05). The V_5Gy_ (%) and V_10Gy_ (%) for the ipsilateral lung turned out to be similar between VMAT and TSP techniques.

**Conclusions:**

Our study indicates that VMAT should be a better choice of radiotherapy for left-sided breast cancer patients after radical mastectomy. If VMAT is unavailable, 9FIMRT can achieve better CI and HI values and be more MU-efficient compared with TSP; however, TSP can effectively reduce the low dose volume of the ipsilateral lungs and heart.

## 1. Introduction

**Figure 1:**
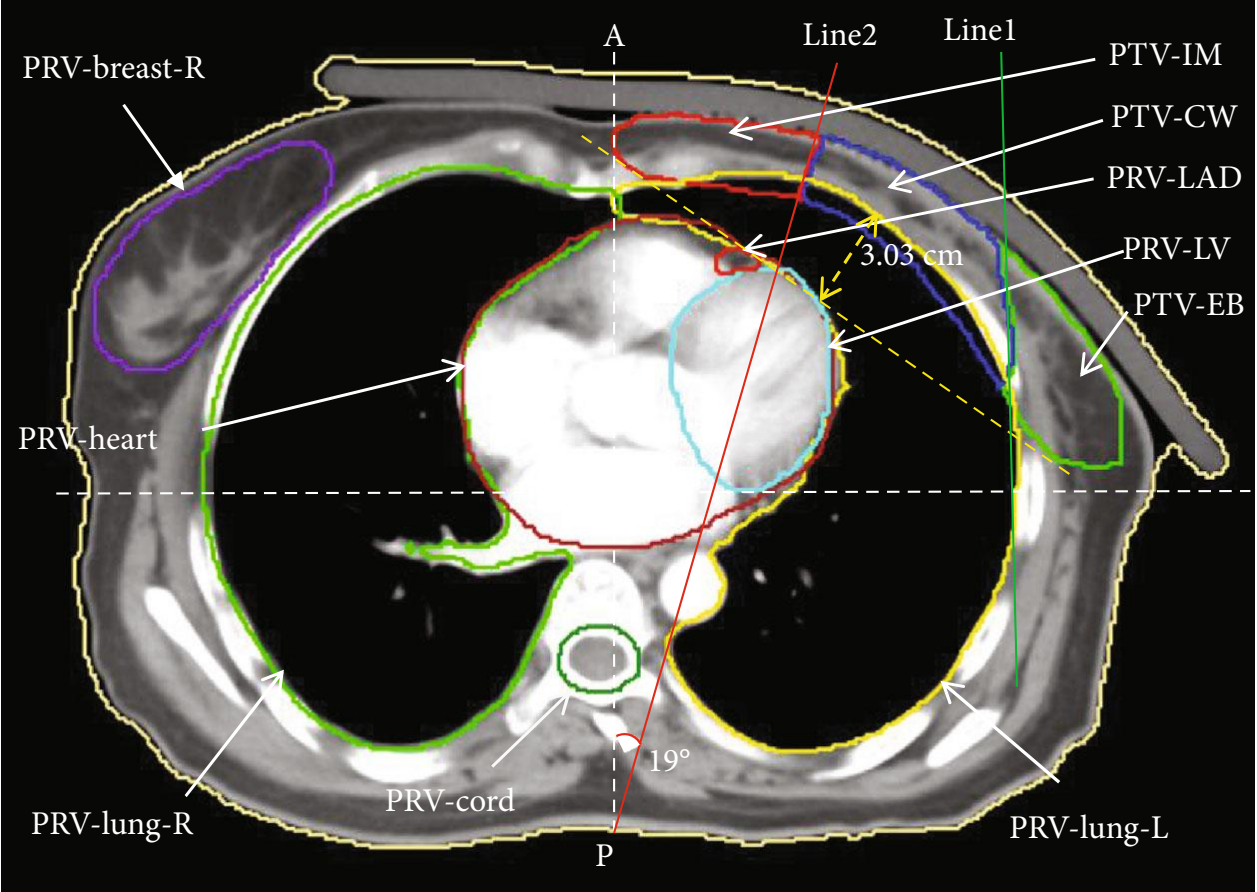
Targets segmental setting of TSP in an axial view of CT. Abbreviations: PTV-CW: chest wall region of PTV (blue); PTV-IM: internal mammary nodes region of PTV (red); PTV-EB: external breast region of PTV (green).

Breast cancer is the most common cancer in females worldwide [1]. Radiotherapy after radical mastectomy is an important treatment modality for the patients with advanced breast cancer, which can significantly reduce the recurrence rate and improve the survival rate [2–4]. Due to individual anatomical variation, we sometimes see breast cancer patients with a large chest wall curvature (e.g., Figure 1), which in our clinic was quantified with the maximum distance between PTV's tangent and the outermost of the ipsilateral lung being more than 3 cm (e.g., Figure 1). The shape of the irradiation target is irregular, concave, and very patient-specific. Meanwhile, the adjacent organs at risk (OARs) including the ipsilateral lung and heart make planning difficult for patients. At present, the main methods of postoperative radiotherapy for patients with advanced breast cancer include three-dimensional conformal radiotherapy (3DCRT), intensity modulated radiotherapy (IMRT), volumetric modulated arc therapy (VMAT), and the combination of 3DCRT and IMRT [5–9]. The selection of the optimal radiation-delivery technique remains a critical component to individualize the breast cancer treatment, which requires adequate dose coverage as well as OARs sparing for each patient's unique anatomy. Compared to IMRT/VMAT plans, 3DCRT plans tend to have inferior targets coverage, poorer dose conformity, and higher volume of 20 Gy irradiation [10]. The IMRT and VMAT techniques for treating chest wall and regional nodes as a whole PTV after modified radical mastectomy have proven beneficial [5, 6, 9], such as better dose conformity and homogeneity. Compared with the IMRT, VMAT can significantly reduce the treatment time and monitor units while meeting the clinical requirements [5]. However, the application of VMAT technology in China needs to be improved [11]. The prevalent treatment technology for breast cancer in many Chinese hospitals is still IMRT, and the traditional plan setting of 9-field IMRT (9FIMRT) may end up with higher low dose volume inside OARs (ipsilateral lung and heart). In this study, we compared and evaluated the TSP, 9FIMRT, and VMAT techniques for selected left-sided breast cancer patients after radical mastectomy.

## 2. Methods

### 2.1. Ethics Statement

Ethics approval of this case report was granted by the Institutional Ethics Review Board of Renmin Hospital of Wuhan University. A written informed consent was obtained from the patient for publication of this case report and any accompanying images. Institutional approval was not required to publish this manuscript.

### 2.2. Patient Enrollment

A total of 15 breast cancer patients after radical mastectomy were enrolled into this dosimetric planning study. The enrolled patient age ranges from 35 to 66 years old. All the selected patients had radical mastectomy (T3-4 and/or metastatic axillary lymph nodes ≥ 3). The treatment target includes the ipsilateral chest wall, supra/infraclavicular, partial axillary lymph nodes at high risk, and internal mammary nodes (IMN). All the patients have barrel-shaped chest or large anterior chest wall curvature, i.e., the maximum distance of PTV's tangent to the outermost side of the affected lung is more than 3 cm. Figure 1 shows an exemplary patient axial CT image with this anatomic feature.

### 2.3. CT Simulation and CTV/PTV, PRV-OARs Generated

All patients were placed head first and supine position on carbon fiber immobilization board and vacuum bag (Klarity Corporation, Guangzhou, China), with hands holding the respective ipsilateral pole overhead and head turning to the contralateral side. A planning CT scan of 5-mm slice thickness and then reconstructed into 3 mm slice thickness from midneck to diaphragm without contrast enhancement was obtained for each patient using a GE-HiSpeed CT simulator (GE Healthcare, USA). During simulation, 1 cm thick tissue-equivalent bolus (position recorded by marker pen) was placed on the patient's chest wall to enhance the skin dose. Physicians could discontinue the use of bolus at any time according to the skin reaction during radiotherapy, and the updated plan without bolus will be generated. In this study, the boluses of all patients were used throughout the course of treatment without interruption. CTV and OARs (lung, heart, spinal cord, contralateral breast, and humeral heads) were delineated by one specialized radiation oncologist according to the Breast Cancer Atlas [12] of the Radiation Therapy Oncology Group (RTOG). Because of uncertainties and variation in the position of the OAR during treatment, the PRV contours of all the involved OARs were outlined by the same specialized radiation oncologist. According to different OARs, PRV-OARs were added a 1 to 3 mm expansion in all directions around the OARs. Considering the systematic and random setup errors in the treatment process in our department, the planning target volume (PTV) was generated from CTV with uniform 5 mm margin, taking into account the effect of respiratory movement. Zhang et al. [13] evaluated the intrafraction motion of the chest wall and found that the maximum displacement was around 3 mm. We used 4DCT to observe the range of motion of the chest wall and found similar results, so we take PTV + 5 mm in the anterior direction as the optimized target structure. This will enable the end of MLC to cover the respiratory movement, so-called “skin flash,” and keep the PTV 3 mm away from the bolus included body contour. CT images and contours were transferred to the Philip TPS (Pinnacle^3R^ v9.10). All plans were generated from Pinnacle system.

### 2.4. TSP Definition and Planning

#### 2.4.1. TSP Definition

In TSP, PTV was divided into four regions: supra/infraclavicular region (PTV-SC), internal mammary nodes region (PTV-IM), chest wall region (PTV-CW), and external breast region (PTV-EB) as shown in Figure 1. The definition of PTV-SC is the same as that of RTOG Breast Cancer Atlas [11]. The other three regions are determined by dividing PTV with two lines in each axial slice. The first line is a vertical line tangential to the most lateral edge of the ipsilateral lung. The second line passes through the internal mammary artery 7-10 mm lateral to the midline in the anterior-posterior direction (AP), and the angle between this line and AP direction was approximately 10° to 20° depending on patient anatomy. These two lines divide the original PTV (excluding PTV-SC) into PTV-IM (medial), PTV-CW (intermediate), and PTV-EB (lateral). The boundaries of the four segments are described in Table 1.

#### 2.4.2. TSP Planning

**Figure 2:**
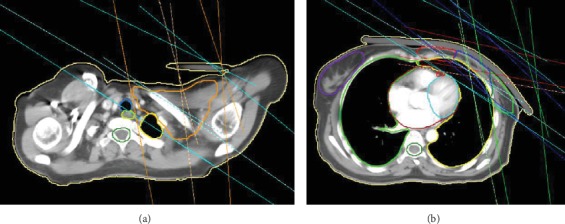
(a) Beam view of PTV-SC and PTV. (b) Beam view of the PTV exclude the PTV-SC. Abbreviations: Beam of PTV: cyan; Beam of PTV-SC: orange; Beam of PTV-IM: red; Beam of PTV-CW: blue; Beam of PTV-EB: green.

TSP uses 9 IMRT fields with single isocenter at the center of mass of the PTV showed in Figure 2. A single isocenter could avoid the possible intrafractional deviation and field matching complexity caused by multiple isocenter treatment. The beam angles of the two fields for PTV-SC were arranged to avoid the spinal cord and the humeral head with half-beam block inferiorly (Figure 2(a)). Two half-beam tangential fields are used for PTV-CW. We recommend that, in each axial view, the line of intersection of the two lines and the chest wall has no overlap with the contralateral breast, and the maximum distance to the outermost side of the affected lung was less than 2 cm. Then one field is used for PTV-IM with beam angled about 100° to avoid heart tissue as much as possible. Two half-beam fields are used for PTV-EB by setting the beam angles to keep them from lung tissue as far as possible. The other two fields are added to increase the PTV dose homogeneity and conformity. The jaw of every field was set to fit each region (PTV-CW, IM, and EB) as shown in Figure 2(b), and the overlap distance of jaws in the superior-inferior direction were about 1-2 cm from our planning experience, to avoid the hot or cold dose points near the segmented regions. The collimator angle is set so that the MLC moves perpendicular to the long axis of the segmented target.

#### 2.4.3. 9FIMRT Planning

Conventional IMRT plan had the same number of fields as TSP in order to reduce the deviation caused by different number of fields. Each conventional IMRT plan uses the same isocenter as its corresponding TSP and employs totally 9 coplanar irradiation fields among which two were tangential fields with coincident lower field edges using half-field technique. Based on these two tangential fields, three more fields were added with gantry angles increased every 10° from the medial tangential beam toward anterior direction, another three fields every 10° from the lateral tangential beam towards anterior direction and 0° beam. The jaws of all fields were fixed to fit the whole PTV instead of to each individual the segmental region, and the collimator angle was set to have the MLC move perpendicularly to the long axis of the PTV.

Direct machine parameter optimization (DMPO) was applied to optimize TSP and 9FIMRT plans, and jaw motion was not allowed. The max iterations were 100, and the convolution dose iteration was 40. The minimum field size and monitor unite of subfield were restricted as 5 cm^2^ and 5 MU, respectively. The two plans were delivered using step and shoot technique with a dose rate of 600 MU/min.

### 2.5. VMAT Planning

The VMAT plans were consisted of two arcs rotating from 290-310 to 160-180 degrees and reversely with collimator setting of 15 degrees. The VMAT plans were generated by the same planner on the same target and assistant contours in Pinnacle^3R^ system (v 9.10). The optimization was Smart Arc, and dose distribution was calculated with convoluted collapsed cone algorithm with 3 mm dose grid resolution and 4° control point spacing. The jaw tracking function was activated.

### 2.6. Plan Optimization and Dose criteria [10, 14]

In TSPs, all segmental targets were given the same objectives and cooptimized together. All plans were normalized so that D_95_ of the PTV = 50 Gy and shared the same optimization objectives of dose volume histogram (DVH) as follows:

(1) PTV: V_50Gy_ ≥ 95%, V_55Gy_ < 15%, V_57.5Gy_ < 5%, and V_47.5Gy_ ≥ 98%
(2) OARs:
  - Ipsilateral lung: D_mean_ ≤ 15 Gy, V_5Gy_ ≤ 60%, V_10Gy_ ≤ 40%, V_20Gy_ ≤ 30%, and V_30Gy_ ≤ 20%
  - Contralateral lung: V_5Gy_ ≤ 20%.
  - Contralateral breast: D_max_ ≤ 40 Gy and D_mean_ ≤ 3 Gy
  - Spinal cord: D_max_ ≤ 45 Gy
  - Humeral head: D_mean_ ≤ 50 Gy
  - Heart: D_mean_ ≤ 8 Gy, V_20Gy_ ≤ 10%, and V_30Gy_ ≤ 5%

### 2.7. PTV and OARs Dose Comparison among TSP, 9FIMRT, and VMAT Plans

The PTV evaluation mainly used homogeneity index (HI) and conformity index (CI). We used the HI proposed in ICRU-83 [15]:

(1) $$\begin{array}{c} \text{HI}=\frac{\left(D_{2}-D_{98}\right)}{D_{p}}, \end{array}$$

where D_2_ and D_98_ represent the dose received by 2% and 98% volume of PTV, respectively, and D_p_ is the prescription dose. The CI is defined as [16]

(2) $$\begin{array}{c} \text{CI}=\frac{{TV}_{PIV}^{2}}{TV\times V_{RI}}, \end{array}$$

where TV_PIV_ is the PTV volume covered by the prescription dose, TV is the PTV volume, and V_RI_ is the total volume covered by the prescription dose. Meanwhile, the following parameters of the two planning techniques were compared:

(1) for PTV: D_2%_, D_98%_, and D_mean_
(2) for OARs:
  - for ipsilateral lung: V_5Gy_, V_10Gy_, V_20Gy_, V_30Gy_, and D_mean_
  - for heart: D_mean_, V_5Gy_, V_10Gy_, V_20Gy_, V_30Gy_, D_mean_, LV, and LAD
  - for contralateral breast: D_mean_ and D_max_
  - for spinal cord: D_max_
  - for left humeral head: D_mean_

### 2.8. Statistical Analysis

The results were represented as mean ± standard deviation (SD). The nonparametric Friedman test was used to compare the three plans, and the nonparametric Wilcoxon signed rank test was selected for the comparison between two plans by SPSS 19.0 software (IBM Corp., Armonk, NY, USA); the *p*-value less than 0.05 was considered statistically significant.

## 3. Results

### 3.1. PTV Dose Parameters Comparisons

We have summarized the dosimetric results of PTV in Table 2. The average and standard deviation of the volume of PTV was 958 ± 101 cm^3^. The VMAT plans showed higher CI of PTV than 9FIMRT and TSP plans (0.79 ± 0.02 [VMAT] vs. 0.75 ± 0.03 [9FIMRT] vs. 0.69 ± 0.02 [TSP], *p* < 0.05). Compared with TSP and 9FIMRT, the VMAT plans had the least MU (639 ± 120 [VMAT] vs. 810 ± 129 [9FIMRT] vs. 933 ± 120 [TSP], *p* < 0.05) and shorter delivery time (2.87 ± 0.80 [VMAT] vs. 6.04 ± 0.39 [9FIMRT] vs. 6.14 ± 0.41 [TSP], *p* < 0.05). The HI difference between 9FIMRT and VMAT plans was not statistically significant in this study (*p* > 0.05), and the TSP got worse HI values than 9FIMRT and VMAT (0.20 ± 0.03 [TSP] vs. 0.17 ± 0.03 [9FIMRT] vs. 0.16 ± 0.02 [VMAT], *p* < 0.05).

### 3.2. OARs Dose Parameters Comparisons

Table 3 listed the detailed comparisons of dose parameters of PRVs of the lungs, heart, contralateral breast, spinal cord, and left humeral head for the patients using TSP, 9FIMRT, and VMAT plans.

#### 3.2.1. Ipsilateral Lung Dose Comparison

**Figure 3:**
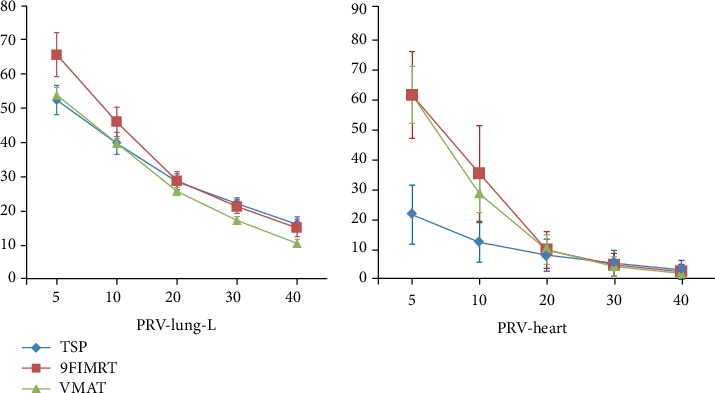
Average dose-volume histogram (DVH) comparison for the ipsilateral lung and heart.

Compared with 9FIMRT plans, except V_20Gy_ (%) (28.45 ± 2.36 [TSP] vs. 28.78 ± 2.66 [9FIMRT], *p* > 0.05), the VMAT and TSP plans had significantly reduced the V_5Gy_, V_10Gy_, and D_mean_ for the ipsilateral lung (*p* < 0.05). Both VMAT and TSP plans showed similar protection to the ipsilateral lung with respect to its V_5Gy_ and V_10Gy_. However, VMAT plans had lower V_20Gy_, V_30Gy_, and V_40Gy_ than those of TSP and 9FIMRT plans (*p* < 0.05).  Figure 3 shows the average DVH parameters. The VMAT plans significantly reduced dose irradiation volume in the ipsilateral lung.

#### 3.2.2. Heart Dose Comparison

The V_5Gy_ (%), V_10Gy_ (%), V_20Gy_ (%), V_30Gy_ (%), and D_mean_ (Gy) of the left ventricle (LV), left anterior descending artery (LAD), and whole heart dose comparison among three techniques were shown in Table 2, respectively. The low-dose irradiated area (V_5Gy_, V_10Gy_, and V_20Gy_) and D_mean_ for the heart were significantly reduced with TSP plans (*p* < 0.05), and the average DVH of the heart was shown in Figure 3. There was no statistical difference in D_mean_ of LAD in the three techniques in our study (*p* > 0.05). However, the D_mean_ of LV in TSP was significantly lower than that in 9FIMRT and VMAT (8.05 ± 4.21 [TSP] vs. 12.78 ± 4.52 [9FIMRT] vs. 9.91 ± 2.86 [VMAT], *p* < 0.05).

#### 3.2.3. Others OARs Dose Comparison

D_mean_ (Gy) to contralateral breast was similar among the three planning techniques (2.91 ± 1.79 [TSP] vs. 3.54 ± 1.48 [9FIMRT] vs. 3.11 ± 0.28 [VMAT]). However, the 9FIMRT got relatively higher D_mean_ than TSP plans (*p* < 0.05, respectively). In our study, we found the VMAT technique performed the best for the protection of D_max_ (Gy) to the contralateral breast (7.39 ± 2.61 [VMAT] vs. 23.45 ± 13.5 [9FIMRT] vs. 28.07 ± 16.46 [TSP], *p* < 0.05). The dosimetric parameters of spinal cord and left humeral head were all within our safe dose criteria.

## 4. Discussion

**Figure 4:**
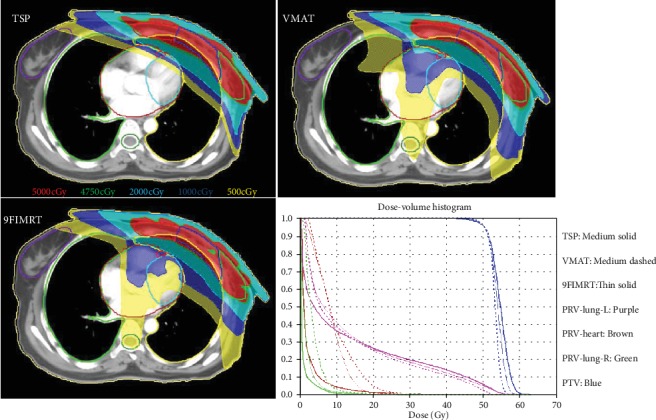
Dose distribution and DVH of three plan techniques.

In our study, all plans met the target coverage and the hot spot dose limit of PTV (V_55Gy_ < 15% and V_57.5Gy_ < 5%), and we found that the TSP showed worse CI and HI compared with VMAT and 9FIMRT. However, TSP showed better protection of the volumes of low dose in the heart and ipsilateral lung as shown in Figure 4. In patients after breast-conserving surgery, conformal radiotherapy (CRT) combined with IMRT can effectively reduce V_5Gy_ and V_10Gy_ of the affected lungs in clinics [17]. Nevertheless, for most patients undergoing radical mastectomy, conformal radiotherapy combined with IMRT technique may result in large V_20Gy_ in the lung of the affected side and therefore should be avoided if possible. Especially in the case of IMN-involved target, with the increase of the distance from axillary line, the entire target becomes more concave, which greatly increases the difficulty of treatment planning. In this study, the average DVH's parameters of the ipsilateral lungs and heart were shown in Figure 3, which indicated that the TSPs have a better protection to the ipsilateral lungs and heart compared with 9FIMRT. We believe that in the radiotherapy for the left-sided breast cancer patients, the limited reduction of target dose homogeneity is a worthwhile trade-off for smaller low dose volumes in the heart and ipsilateral lung [18].

Radiation-induced pneumonitis risk is an important radiotherapy complication in breast cancer patients after radiotherapy [19, 20]. Dosimetry parameters influencing the radiation induced pneumonitis risk conventionally include V_5Gy_, V_10Gy_, and V_20Gy_. However, it is still controversial which one(s) is a better predictor. Caudell et al. [17] and Gopal et al. [21] concluded that there is a positive correlation between V_20Gy_ and the radiation-induced pneumonitis risk in the affected lung. Willner et al. [22] suggested that the incidence of radiation induced pneumonitis increased by 10% for every 10% increase in V_10Gy_. Yorke et al. [23] proposed that V_5Gy_ and V_10Gy_ of the affected lung may be effective predictors of radiation-induced lung injury. In this study, all low dose parameters of ipsilateral lung (including D_mean_, V_10Gy_, and V_5Gy_) are significantly smaller in VMAT and TSP plans compared with those in the corresponding 9FIMRT plans. The VMAT and TSP can significantly reduce the low radiation dose and volume of the affected side of the lung while ensuring sufficient irradiation to the target area, which may reduce the incidence of radiation-induced lung injury.

With the advancement of technology, VMAT has been gaining popularity in radiation therapy. The number of accelerators per million people in China is much lower than the average level in developed countries [11]. Higher efficiency treatment technique could benefit more cancer patients in China, on the premise of ensuring the quality of treatment. Therefore, the implementation rate of VMAT technology in China needs to be improved. Some studies compared VMAT and other techniques for the radiation therapy after radical resection of breast cancer. For example, Lai et al. [10] compared 3DCRT with three VMATs planning techniques (conventional VMAT, modified VMAT, and modified VMAT using FFF beams) and found that the modified VMAT using FFF beams could result in the highest ipsilateral lung's V_5Gy_ (70.3 ± 5.8%). Zhang et al. [13] compared the step and shoot IMRT with the conventional VMAT, and their results show that VMAT is superior to static IMRT regarding the dosimetric parameters for both PTV and OARs which could be related to the beam gantry angle combination, i.e., 300°, 0°, 40°, 80°, and 110°. Ma et al. [5] also compared the 3DCRT with field-in-field technique (3DCRT-FinF), 5-field IMRT, and 2-partial-arc VMAT. The V_5Gy_, V_10Gy_, and V_20Gy_ of 5-field IMRT plans and 2VMAT plans were 52.53 ± 7.65% vs. 70.36 ± 8.84%, 36.89 ± 7.75% vs. 51.67 ± 8.72%, and 27.77 ± 7.08% vs. 34.08 ± 7.16%, respectively. The 5-field IMRT plans performed better than 2-partial-arc VMAT plans. The use of VMAT by Lai et al. [10] resulted in a smaller volume of high dose in the lung (V_20Gy_) but larger lung volume with low dose (V_5Gy_). In contrast, Ma et al. [5] used static 5-field IMRT techniques achieved smaller low dose volumes (i.e., V_5Gy_ and V_10Gy_) but larger high dose volume (V_20Gy_). Ma et al. [5] also found that 3DCRT-FinF technique had similar dosimetric result for ipsilateral lung compared to our study. Nevertheless, their 3DCRT-FinF plans got inadequate targets coverage (V_95%_ was 78.23 ± 4.25%) and poor dose conformity (CI was 0.27 ± 0.07). In our study, VMAT plans had the best CI and HI values, the superior protection to the ipsilateral lungs and lower D_max_ of contralateral breast. Meanwhile, TSP got the best protection to the heart. In summary, there is no standard radiotherapy treatment planning technique for breast cancer after radical mastectomy yet, and diverse choices employing different technologies are available.

Breast cancer radiotherapy has an impact on the contralateral breast as well. Popescu et al. [24] used RapidArc® technique and reported that the contralateral breast D_mean_ less than 3.2 Gy, which could significantly reduce the risk of secondary carcinogenesis caused by radiation therapy, especially for young female patient. For all the plans created in this study, the D_mean_ of contralateral breast is close to that reported by Popescu et al. [24] (2.91 ± 1.79 Gy [TSP], 3.54 ± 1.48 Gy [9FIMRT] vs. 3.11 ± 0.28 Gy [VMAT]). Moreover, VMAT plans had the lowest D_max_ of contralateral breast.

Darby et al. [25] reported a linear relationship between ischemic heart disease and D_mean_ to the heart. The incidence of coronary events increased by 7.4% per Gray mean dose to the heart relatively. Except for V_30Gy_, the TSP showed lower dosimetric results of the heart than VMAT and 9FIMRT. D_mean_ of the heart dropped from 9.92 ± 2.76 Gy for 9FIMRT and 9.31 ± 1.62 Gy for VMAT to 5.39 ± 2.45 Gy for TSP (*p* < 0.05). The dose of the left anterior descending artery and left ventricle was generally greater than the dose to the whole heart during the radiation treatment course. Taylor et al. [26] found an increase in myocardial perfusion defects in the region supplied by the left anterior descending artery 6 months after it was irradiated. The data of Kaidar-Person et al. [27] suggested that irradiation of the left ventricle can result in early post-RT perfusion defects and there appeared to be a strong dose/volume dependence to the risk. In our study, three techniques showed similar mean dose of left anterior descending artery (*p* > 0.05). The D_mean_ of the left ventricle for TSPs was smaller than the others (*p* < 0.05). It seems that the TSP technique has the potential to reduce the incidence of ischemic heart disease caused by radiotherapy by bringing down the D_mean_ of the heart and LV.

Fewer beam delivery time and MUs can reduce the risk of patient intrafraction motion as well as the scattered dose to the patients, which subsequently mitigate the probability of long-term secondary carcinogenesis in patients [28]. We used Arccheck (Sun Nuclear Corp., Melbourne, FL, USA) in QA mode to record delivery time of different techniques and noticed that the VMAT has a big advantage compared with the other two techniques with regard to total MUs and delivery time, which is only about 68% (MUs) and 46% (DT) of those of TSP. Although the number of total MUs and DT of TSP is the largest, there was a little difference in the absolute values compared with 9FIMRT.

## 5. Conclusions

In summary, the VMAT technique in this study demonstrated the superior dose conformity and homogeneity to the 9FIMRT and TSP while ensuring enough prescribed dose to the target of radiation therapy. It also significantly reduce the risk of complication of the ipsilateral lung and contralateral breast with lower dose radiation exposure for breast cancer patients. Although there are some disadvantages in the CI, HI, and MU for TSPs, it greatly reduces the dose of the ipsilateral lung and heart, especially the mean dose of the heart and left ventricle. We recommend that TSP is more worthy of clinical choice than 9FIMRT in the absence of VMAT.

## Figures and Tables

**Table 1:** Anatomic borders of divided PTV regions in TSP.

Structures	Anterior	Posterior	Medial	Lateral	Cranial	Caudal
PTV-SC	Same as supra/infraclavicular border of RTOG					
PTV-EB	Posterior border of PTV	Posterior border of PTV	Vertical line of the maximum spacing between of lungs	Lateral border of PTV	Caudal border of PTV-SC	Caudal border of PTV
PTV-CW	Anterior border of PTV	Posterior border of PTV	Lateral border of PTV-IM	Medial border of PTV-EB	Caudal border of PTV-SC	Caudal border of PTV
PTV-IM	Anterior border of PTV	Posterior border of PTV	Medial border of PTV	7-10 mm margin of internal mammary vessels	Caudal border of PTV-SC	Caudal border of contralateral breast

Abbreviations: PTV: planning target volume; PTV-SC: supra/infraclavicular PTV region; PTV-CW: chest wall PTV region; PTV-IM: internal mammary nodes PTV region; PTV-EB: external breast PTV region; TSP: target segmented planning; RTOG: Radiation Therapy Oncology Group.

**Table 2:** PTV dose parameters of three plans in left-sided breast cancer patients with postmastectomy (*x* ± s).

Technique	No.	D_2%_ (Gy)	D_98%_ (Gy)	D_mean_ (Gy)	HI	CI	MU	DT (min)
TSP	15	58.41 ± 1.37	48.25 ± 0.41	54.14 ± 0.78	0.20 ± 0.03	0.69 ± 0.02	933 ± 120	6.14 ± 0.41
9FIMRT	15	57.38 ± 1.14^a^^∗^	48.77 ± 0.52^a^^∗^	53.62 ± 0.61^a^^∗^	0.17 ± 0.03^a^^∗^	0.75 ± 0.03^a^^∗^	810 ± 129^a^^∗^	6.04 ± 0.39^a^^∗^
VMAT	15	56.64 ± 0.63^b^^∗^	48.84 ± 0.41^b^^∗^	53.52 ± 0.54^b^^∗^	0.16 ± 0.02^b^^∗^	0.79 ± 0.02^b^^∗^^, c^^∗^	639 ± 120^b^^∗^^, c^^∗^	2.87 ± 0.80^b^^∗^^, c^^∗^

Abbreviations: a: 9FIMRT vs. TSP; b: VMAT vs. TSP; c: VMAT vs. 9FIMRT; ∗*p* < 0.05; TSP: target segmented planning; 9FIMRT: 9 fields IMRT; VMAT: volumetric modulated arc therapy; DT: delivery time.

**Table 3:** OARs' dose parameters of the three plans in left-sided breast cancer patients with postmastectomy (*x* ± s).

Technique	No.	PRV-lung-L				PRV-LAD	PRV-LV	PRV-cord
		V_5Gy_	V_10Gy_	V_20Gy_	D_mean_	D_mean_	D_mean_	D_max_
TSP	15	52.44 ± 4.31	39.71 ± 3.23	28.45 ± 2.36	14.88 ± 0.85	37.47 ± 12.12	8.05 ± 4.21	24.23 ± 12.38
9FIMRT	15	65.76 ± 6.49^a^^∗^	46.01 ± 4.35^a^^∗^	28.78 ± 2.66	16.52 ± 1.45^a^^∗^	38.21 ± 11.69	12.78 ± 4.52^a^^∗^	28.61 ± 8.41
VMAT	15	53.91 ± 2.23^b^^∗^^, c^^∗^	39.84 ± 1.25^c^^∗^	25.64 ± 1.19^b^^∗^^, c^^∗^	13.88 ± 0.51^b^^∗^^, c^^∗^	37.05 ± 9.67	9.91 ± 2.86^b^^∗^^, c^^∗^	34.93 ± 7.41^b^^∗^^, c^^∗^

Technique	No.	PRV-heart				PRV-breast-R		PRV-hum-head-L
		V_5Gy_	V_10Gy_	V_20Gy_	D_mean_	D_mean_	D_max_	D_mean_
TSP	15	21.37 ± 9.62	12.06 ± 6.65	7.76 ± 5.35	5.39 ± 2.45	2.91 ± 1.79	28.07 ± 16.46	40.94 ± 6.38
9FIMRT	15	61.46 ± 14.52^a^^∗^	35.15 ± 16.01^a^^∗^	9.48 ± 6.14^a^^∗^	9.92 ± 2.76^a^^∗^	3.54 ± 1.48^a^^∗^	23.45 ± 13.5	41.84 ± 5.08^a^^∗^
VMAT	15	61.52 ± 9.55^b^^∗^	28.37 ± 6.31^b^^∗^	9.64 ± 4.94^b^^∗^	9.31 ± 1.62^b^^∗^	3.11 ± 0.28	7.39 ± 2.61^b^^∗^^, c^^∗^	40.35 ± 4.75

Abbreviations: a: 9FIMRT vs. TSP; b: VMAT vs. TSP; c: VMAT vs. 9FIMRT; ^∗^*p* < 0.05. LAD: left anterior descending artery; LV: left ventricle.

## Data Availability

The data used to support the findings of this study are available from the corresponding author upon request.

## Abbreviations

TSP: Target segmented planning
IMRT: Intensity modulated radiotherapy
CTV: Clinical target volume
PTV: Planning target volume
IMN: Internal mammary nodes
OARs: Organs at risk
VMAT: Volumetric modulated arc therapy
RTOG: Radiation Therapy Oncology Group
PTV-SC: Supra/infraclavicular PTV region
PTV-CW: Chest wall PTV region
PTV-IM: Internal mammary nodes PTV region
PTV-EB: External breast PTV region
DVH: Dose-volume histograms
HI: Homogeneity index
CI: Conformity index
PR: Pneumonitis risk
MU: Monitor units
LAD: Left anterior descending artery
LV: Left ventricle
DT: Delivery time.

## References

[1] WHO *Fact Sheets by Cancer*.

[2] American Cancer Society *Radiation for Breast Cancer*.

[3] Van de Steene J., Soete G., Storme G. (2000). Adjuvant radiotherapy for breast cancer significantly improves overall survival: the missing link. *Radiotherapy and Oncology*.

[4] Onitilo A. A., Engel J. M., Stankowski R. V., Doi S. A. (2015). Survival comparisons for breast conserving surgery and mastectomy revisited: community experience and the role of radiation therapy. *Clinical Medicine & Research*.

[5] Ma C., Zhang W., Lu J. (2015). Dosimetric comparison and evaluation of three radiotherapy techniques for use after modified radical mastectomy for locally advanced left-sided breast cancer. *Scientific Reports*.

[6] Schubert L. K., Gondi V., Sengbusch E. (2011). Dosimetric comparison of left-sided whole breast irradiation with 3DCRT, forward-planned IMRT, inverse- planned IMRT, helical tomotherapy, and topotherapy. *Radiotherapy and Oncology*.

[7] Lin J. F., Yeh D. C., Yeh H. L., Chang C. F., Lin J. C. (2015). Dosimetric comparison of hybrid volumetric-modulated arc therapy, volumetric-modulated arc therapy, and intensity-modulated radiation therapy for left-sided early breast cancer. *Medical Dosimetry*.

[8] Jin G. H., Chen L. X., Deng X. W., Liu X. W., Huang Y., Huang X. B. (2013). A comparative dosimetric study for treating left-sided breast cancer for small breast size using five different radiotherapy techniques: conventional tangential field, filed-in-filed, tangential-IMRT, multi-beam IMRT and VMAT. *Radiation Oncology*.

[9] Yang B., Wei X. D., Zhao Y. T., Ma C. M. (2014). Dosimetric evaluation of integrated IMRT treatment of the chest wall and supraclavicular region for breast cancer after modified radical mastectomy. *Medical Dosimetry*.

[10] Lai Y., Chen Y., Wu S. (2016). Modified volumetric modulated arc therapy in left sided breast cancer after radical mastectomy with flattening filter free versus flattened beams. *Medicine*.

[11] Lang J., Wang P., Dake W. U. (2016). An investigation of the basic situation of radiotherapy in mainland China in 2015. *Chinese Journal of Radiation Oncology*.

[12] Radiation Therapy Oncology Group (RTOG) Breast Cancer Contouring Altas. http://www.rtog.org/CoreCab/ContouringAtlases/BreastCancerAtlas.aspx.

[13] Zhang Q., Yu X. L., Hu W. G. (2015). Dosimetric comparison for volumetric modulated arc therapy and intensity-modulated radiotherapy on the left-sided chest wall and internal mammary nodes irradiation in treating post-mastectomy breast cancer. *Radiology and Oncology*.

[14] Ma J., Li J., Xie J. (2013). Post mastectomy linac IMRT irradiation of chest wall and regional nodes: dosimetry data and acute toxicities. *Radiation Oncology*.

[15] Hodapp N. (2012). The ICRU Report 83: prescribing, recording and reporting photon-beam intensity-modulated radiation therapy (IMRT). *Strahlentherapie Und Onkologie*.

[16] Paddick I. (2000). A simple scoring ratio to index the conformity of radiosurgical treatment plans. *Journal of Neurosurgery*.

[17] Caudell J. J., de Los Santos J. F., Keene K. S. (2007). A dosimetric comparison of electronic compensation, conventional intensity modulated radiotherapy, and tomotherapy in patients with early-stage carcinoma of the left breast. *International Journal of Radiation Oncology, Biology, Physics*.

[18] Miao J., Yan H., Tian Y. (2017). Reducing dose to the lungs through loosing target dose homogeneity requirement for radiotherapy of non small cell lung cancer. *Journal of Applied Clinical Medical Physics*.

[19] Goldman U. B., Wennberg B., Svane G., Bylund H., Lind P. (2010). Reduction of radiation pneumonitis by V_20_-constraints in breast cancer. *Radiation Oncology*.

[20] Wen G., Tan Y. T., Lan X. W. (2017). New clinical features and dosimetric predictor identification for symptomatic radiation pneumonitis after tangential irradiation in breast cancer patients. *Journal of Cancer*.

[21] Gopal R., Tucker S. L., Komaki R. (2003). The relationship between local dose and loss of function for irradiated lung. *International Journal of Radiation Oncology, Biology, Physics*.

[22] Willner J., Jost A., Baier K., Flentje M. (2003). A little to a lot or a lot to a little? An analysis of pneumonitis risk from dose-volume histogram parameters of the lung in patients with lung cancer treated with 3-D conformal radiotherapy. *Strahlentherapie und Onkologie*.

[23] Yorke E. D., Jackson A., Rosenzweig K. E., Braban L., Leibel S. A., Ling C. C. (2005). Correlation of dosimetric factors and radiation pneumonitis for non-small-cell lung cancer patients in a recently completed dose escalation study. *International Journal of Radiation Oncology, Biology, Physics*.

[24] Popescu C. C., Olivotto I. A., Beckham W. A. (2010). Volumetric modulated arc therapy improves dosimetry and reduces treatment time compared to conventional intensity-modulated radiotherapy for locoregional radiotherapy of left-sided breast cancer and internal mammary nodes. *International Journal of Radiation Oncology, Biology, Physics*.

[25] Darby S. C., Ewertz M., McGale P. (2013). Risk of ischemic heart disease in women after radiotherapy for breast cancer. *The New England Journal of Medicine*.

[26] Taylor C. W., Nisbet A., McGale P., Darby S. C. (2007). Cardiac exposures in breast cancer radiotherapy: 1950s-1990s. *International Journal of Radiation Oncology•Biology•Physics*.

[27] Kaidar-Person O., Meattini I., Jain P. (2017). Discrepancies between biomarkers of primary breast cancer and subsequent brain metastases: an international multicenter study. *Breast Cancer Research and Treatment*.

[28] Ravichandran R., Al-Kindi F., Davis C. A. (2013). Dosimetric comparison of intensity modulated radiotherapy isocentric field plans and field in field (FIF) forward plans in the treatment of breast cancer. *Journal of Medical Physics*.

